# Female Fertility Has a Negative Relationship With Longevity in Chinese Oldest-Old Population: A Cross-Sectional Study

**DOI:** 10.3389/fendo.2020.616207

**Published:** 2021-02-03

**Authors:** Qiao Zhu, Shihui Fu, Qian Zhang, Jinwen Tian, Yali Zhao, Yao Yao

**Affiliations:** ^1^ Central Laboratory, Hainan Hospital of Chinese People’s Liberation Army General Hospital, Sanya, China; ^2^ Department of Cardiology, Hainan Hospital of Chinese People’s Liberation Army General Hospital, Sanya, China; ^3^ Department of Neurology, Hainan Hospital of Chinese People’s Liberation Army General Hospital, Sanya, China; ^4^ Center for Healthy Aging and Development Studies, Raissun Institute for Advanced Studies, National School of Development, Peking University, Beijing, China; ^5^ Center for the Study of Aging and Human Development and Geriatrics Division, Medical School of Duke University, Durham, NC, United States

**Keywords:** female fertility, oldest-old population, centenarian, longevity, negative relationship

## Abstract

**Background:**

Despite research efforts in this field for more than a century, the relationship between female fertility and longevity is unclear. This study was designed to investigate this relationship in Chinese oldest-old population.

**Methods:**

The China Hainan Centenarian Cohort Study was performed in 18 cities and counties of Hainan. A total of 1,226 females, including 758 centenarian women and 468 women aged 80–99 years, were enrolled in this study. Using a standardized protocol, in-person interviews and blood analyses were conducted by a well-trained research team through home visits.

**Results:**

Centenarian women had significantly lower number of children (NOC) and higher initial childbearing age (ICA) and last childbearing age (LCA) than women aged 80–99 years (p < 0.05 for all). Multivariate logistic regression analysis showed that NOC and testosterone (T) levels were positively associated with women aged 80–99 years, when centenarian women was considered as reference (p < 0.05 for all). ICA, LCA, and estradiol (E2) levels were negatively associated with women aged 80–99 years, when centenarian women was considered as reference (p < 0.05 for all).

**Conclusions:**

The centenarians had crucial characteristics of less and delayed childbearing, indicating a negative relationship between female fertility and longevity in Chinese oldest-old population. Serum E2 levels were positively associated and serum T levels were negatively associated with longevity. The less and late childbearing might be a significant factor of longevity, and successful aging might be promoted by reducing and delaying female childbearing.

## Introduction

Antagonistic pleiotropy and disposable soma theories have suggested that female fertility is inversely related to life span because there is a metabolic trade-off between reproduction and longevity (1, 2). Disposable soma theory has suggested that aging is a dynamic process with two aspects: random damage and repair of deoxyribonucleic acid in the process of life (3, 4). The human body is affected by a variety of external and internal damages; aging is accompanied by accumulated damage and continuous repair. When the repair cannot make up for the damage due to the energy shortage caused by growth and reproduction, the body will eventually die (5). Meanwhile, antagonistic pleiotropy theory has suggested that the gene has multiple functions, some of which are beneficial to growth and reproduction and some of which are not (6). Evolutionary selection of mutated genes helps people grow and reproduce when they are young, but these genes lead people to age with time because the main function has been achieved, i.e., passing genetic information on to the next generation (7). Previous studies have shown a negative relationship between female fertility and life span because of the high energy expenditure in female reproduction (8–13). In the Caerphilly study, female fertility has been found to be negatively correlated with post-reproductive longevity (14, 15). However, other studies have found no significant relationship between female fertility and life span (16, 17). A contemporary study involving Norwegians have also highlighted a positive relationship between female fertility and post-reproductive longevity (18, 19). Despite research efforts in this field for more than a century, this relationship is unclear. Hence, there is a need for studies clarifying the relationship between female fertility and longevity.

Centenarians represent a prototype of successful aging, and centenarian women experience an appropriate pattern of fertility (20–23). It might be worth noting the characteristics of female fertility in the centenarians to identify ways to promote successful aging and human longevity. There is growing interest in female fertility in centenarians. To our knowledge, no study has explained the relationship between female fertility and longevity, especially in Chinese oldest-old population (24). Hainan is an area with the highest population density of centenarians in China. The China Hainan Centenarian Cohort Study (CHCCS), with a considerable sample size, provides a significant population-based sample of Chinese centenarians (25). This study was designed to investigate the relationship between female fertility and longevity in Chinese oldest-old population.

## Methods

### Study Population

As a population-based study, the CHCCS was carried out in 18 cities and counties of Hainan, China, from 2014 to 2017. Its cohort profile has been described previously (25). In brief, a total of 1,226 females, including 758 centenarian women and 468 women aged 80–99 years, were enrolled in this study. All participants were naturally aging and never took hormone drugs. This study was approved by the Ethics Committee of Hainan Hospital of Chinese People’s Liberation Army General Hospital (Sanya, Hainan; Number: 301hn11201601). Written informed consent was obtained from each participant prior to the start of this study.

### Standard Procedures

Using a standardized protocol, in-person interviews and blood analyses were conducted by a well-trained research team at Chinese People’s Liberation Army General Hospital through home visits. This interdisciplinary research team included internists, geriatricians, cardiologists, endocrinologists, nephrologists, and nurses. Ethnicity was categorized as either ethnic Han (the predominant ethnicity in China) or others. Education was categorized as illiterate or literate, where illiterate indicated the inability to read and write. Fertility status and age were provided by the participants themselves or friends and relatives of the advanced age, and verified by the number and age of offspring and related population records and medical information. Number of children (NOC) referred to the number of offspring born alive. Initial childbearing age (ICA) referred to the age at which the woman gave birth to the first live offspring. Last childbearing age (LCA) referred to the age at which the woman gave birth to the last live offspring. Barthel Index (BI) was used to evaluate the activities of daily living (ADL); its validity and reliability in Chinese older individuals have been well established (26). BI consists of 10 items that measure a person’s daily activities: grooming, feeding, dressing, bathing, toilet use, transfer from bed to chair, walking, stair climbing, bowel continence, and urinary continence. Each item of BI is rated on a scale with points assigned to each level of activity, and the total score ranges from 0 to 100 points with 5-point increments. A high score indicates a high level of ADL. EuroQol 5 Dimensions (EQ-5D) was used to evaluate the qualities of daily living. EQ-5D covers five dimensions: mobility, self-care, usual activities, pain/discomfort, and anxiety/depression. The standardized index of EQ-5D is based on Chinese data, and validated in various populations with good reliability, with a high score indicating a high quality of daily living (27). Body mass index (BMI) was defined as the weight in kilograms divided by the square of the height in meters. Blood samples were routinely drawn by venipuncture and stored in serum separator tube at 4°C to obtain the serum and transported to the central laboratory in our Biochemistry Department within 4 h. Electrochemiluminescence (Cobas E602; Roche Products Ltd, Basel, Switzerland) was used to determine serum levels of estradiol (E2), testosterone (T), follicle-stimulating hormone (FSH), and luteinizing hormone (LH). Serum levels of total cholesterol (TC), triglyceride (TG), high-density lipoprotein cholesterol (HDL-C), low-density lipoprotein cholesterol (LDL-C), and blood glucose (Glu) were also determined by qualified technicians who were blinded to clinical data.

### Statistical Analyses

EpiData 3.0 software was used to store all the data on a designated computer. Based on α = 0.05 and β = 0.95, the sample size was calculated to be more than 143 individuals for each group. Continuous variables with normal distribution were expressed as mean and standard deviation and compared using student’s t test. Categorical variables were expressed as count and percentage and compared using Chi-square test or Fisher’s exact test. Multivariate logistic regression analysis was carried out to explore fertility status and sex hormones associated with women aged 80–99 years, when centenarian women was considered as reference, after adjusting for ethnicity, education, smoking, drinking, meal times, BI, EQ-5D, BMI, systolic blood pressure (SBP), diastolic blood pressure (DBP), TC, TG, HDL-C, LDL-C, and Glu levels. Statistical analyses were performed with Statistical Package for Social Science (SPSS) software (IBM, NY, USA) and Power Analysis and Sample Size (PASS) software (NCSS, UT, USA). Two-tailed P < 0.05 indicated statistical significance.

## Results

The median age of the study population was 98.28 ± 9.17 years (range: 80–116). As shown in **Table 1**, centenarian women had significantly lower NOC and higher ICA and LCA than women aged 80–99 years (p < 0.05 for all). Serum E2 levels were significantly higher and serum T levels were significantly lower in the centenarian women than in the women aged 80–99 years (P < 0.05 for all). Proportions of illiterate status and smoking were significantly higher, meal times were significantly more, age, SBP, and Glu levels were significantly higher, and BMI, BI, EQ-5D, DBP TC, TG, and LDL-C levels were significantly lower in the centenarian women than in the women aged 80–99 years (P < 0.05 for all). FSH, LH, and HDL-C levels, and proportions of ethnic Han and drinking showed no significant difference between centenarian women and women aged 80–99 years (**Table 1**; P > 0.05 for all).

**Table 1:** Characteristics of oldest-old population aged 80–99 years and ≥100 years.

Characteristics	Total (N = 1226)	80–99 years (N = 468)	≥100 years (N = 758)	P value
Age, years	98.28 ± 9.17	85.55 ± 4.52	102.91 ± 2.87	<0.001
Ethnic Han, n (%)	1,086 (88.6)	414 (88.5)	672 (88.7)	0.927
Illiterate status, n (%)	1,164 (94.9)	432 (92.3)	732 (96.6)	0.001
Smoking, n (%)	65 (5.3)	13 (2.8)	52 (6.9)	0.002
Drinking, n (%)	183 (14.9)	80 (17.1)	103 (13.6)	0.099
Meal times	2.99 ± 0.55	2.96 ± 0.54	3.03 ± 0.57	0.034
BI	81.21 ± 23.33	93.22 ± 13.75	73.79 ± 24.90	<0.001
EQ-5D	0.74 ± 0.23	0.87 ± 0.17	0.67 ± 0.23	<0.001
NOC, n (%)	4.6 ± 2.1	5.2 ± 2.0	4.3 ± 2.2	<0.001
ICA, years	27.6 ± 6.7	25.5 ± 4.5	28.9 ± 7.4	<0.001
LCA, years	41.8 ± 7.4	39.8 ± 5.5	43.0 ± 8.1	<0.001
BMI, kg/m^2^	19.21 ± 3.77	20.68 ± 3.88	18.30 ± 3.39	<0.001
SBP, mmHg	153.30 ± 24.31	151.47 ± 22.89	154.44 ± 25.09	0.034
DBP, mmHg	78.22 ± 13.76	80.97 ± 12.79	76.51 ± 14.39	<0.001
E2, pmol/L	35.50 ± 25.99	25.50 ± 13.39	41.67 ± 29.71	<0.001
T, nmol/L	0.86 ± 1.63	0.90 ± 1.94	0.67 ± 1.35	0.048
FSH, IU/L	83.27 ± 28.90	84.32 ± 37.30	82.60 ± 29.83	0.210
LH, mIU/ml	37.84 ± 17.35	37.13 ± 13.53	38.27 ± 14.82	0.184
TC, mmol/L	4.94 ± 1.09	5.23 ± 1.13	4.76 ± 1.03	<0.001
TG, mmol/L	1.27 ± 0.74	1.35 ± 0.77	1.22 ± 0.71	0.002
HDL-C, mmol/L	1.45 ± 0.41	1.45 ± 0.41	1.46 ± 0.40	0.869
LDL-C, mmol/L	2.97 ± 0.88	3.15 ± 0.97	2.85 ± 0.81	<0.001
Glu, mmol/L	5.00 ± 1.72	4.85 ± 1.90	5.09 ± 1.59	0.016

Data were expressed as mean ± standard deviation or count (percentage).

BI, Barthel Index; EQ-5D, EuroQol 5 Dimensions; NOC, number of children; ICA, initial childbearing age; LCA, last childbearing age; BMI, body mass index; SBP, systolic blood pressure; DBP, diastolic blood pressure; E2, estradiol; T, testosterone; FSH, follicle-stimulating hormone; LH, luteinizing hormone; TC, total cholesterol; TG, triglyceride; HDL-C, high-density lipoprotein cholesterol; LDL-C, low-density lipoprotein cholesterol; Glu, blood glucose.

Multivariate logistic regression analysis showed that NOC and T levels were positively associated with women aged 80–99 years, when centenarian women was considered as reference, suggesting that lower NOC and T levels were significantly associated with longevity, after adjusting for ethnicity, education, smoking, drinking, meal times, BI, EQ-5D, BMI, SBP, DBP, TC, TG, HDL-C, LDL-C, and Glu levels (**Table 2**; p < 0.05 for all). ICA, LCA, and E2 levels were negatively associated with women aged 80–99 years, when centenarian women was considered as reference, suggesting that higher ICA, LCA, and E2 levels were significantly associated with longevity, after adjusting for ethnicity, education, smoking, drinking, meal times, BI, EQ-5D, BMI, SBP, DBP, TC, TG, HDL-C, LDL-C, and Glu levels (**Table 2**; p < 0.05 for all).

**Table 2:** Fertility status and sex hormone associated with women aged 80–99 years in logistic regression analysis with centenarian women as reference.

Variables	B	OR	95% CI	P value
NOC, n (%)	0.288	1.33	1.17–1.53	<0.001
ICA, years	−0.062	0.94	0.91–0.97	<0.001
LCA, years	−0.121	0.89	0.86–0.91	<0.001
E2, pmol/L	−0.045	0.96	0.94–0.97	<0.001
T, nmol/L	0.162	1.18	1.07–1.29	0.001
FSH, IU/L	0.005	1.01	0.99–1.01	0.287
LH, mIU/ml	0.001	1.00	0.98–1.02	0.902

Fertility status and sex hormone associated with women aged 80–99 years in the multivariate logistic regression analysis, when centenarian women were considered as reference, after adjusting for ethnicity, education, smoking, drinking, meal times, BI, EQ-5D, BMI, SBP, DBP, TC, TG, HDL-C, LDL-C, and Glu levels.

OR, odds ratio; CI, confidential interval; NOC, number of children; ICA, initial childbearing age; LCA, last childbearing age; E2, estradiol; T, testosterone; FSH, follicle-stimulating hormone; LH, luteinizing hormone; BI, Barthel Index; EQ-5D, EuroQol 5 Dimensions; BMI, body mass index; SBP, systolic blood pressure; DBP, diastolic blood pressure; TC, total cholesterol; TG, triglyceride; HDL-C, high-density lipoprotein cholesterol; LDL-C, low-density lipoprotein cholesterol; Glu, blood glucose.

## Discussion

Aging is a gradual process whereby the human body loses its ability to physiological repair itself, fertility declines, and the probability of death increases. The evolutionary theories of aging have suggested that the rate of intrinsic aging is associated with a decline in fertility and survival (28). According to disposable soma theory, there is an evolutionary trade-off between fertility and longevity due to the energy requirements for both repair and fertility. However, due to a lack of convincing evidence, the relationships between fertility and longevity have attracted researchers’ attention for more than a century. Some studies have shown a negative correlation between NOC and life span because of the high energy expenditure during the pregnancy and lactation (8–13). Moreover, childbearing requires a lot of physical and mental resources, with detrimental effects on female health and longevity (29–33). Other studies have found no significant correlation between NOC and life span (16, 17). People in several countries or regions take contraception and birth control measures and do not give birth under the conditions of natural fertility, which might make the reproduction-longevity relationship complicated. As an island, Hainan has its unique historical, cultural, social, and economic characteristics: natural birth rates have been very high due to a lack of birth control, and the limited availability of medical resources has led to the limited use of medical interventions. It is worth noting that this presented a natural aging process. There were stable historical and cultural environment, and slow social and economic development. A time lag of at least 10 years between fertile age of women aged 80–99 years and centenarian women has a relatively smaller effect, which gives us an opportunity to explore the relationship between female fertility and life span. Therefore, this study tracked fertility status of women aged over 80 years in Hainan to explore the relationship between female fertility and life span. We found that low NOC had a significant association with longevity, and childbearing might be a harmful factor for longevity, in Chinese oldest-old population.

As per antagonistic pleiotropy theory, undesirable mutations in later life could accumulate if they are beneficial to reproduction at younger age (1). According to disposable soma theory, selective maintenance of somatic cells to promote reproduction and accelerated aging (34). The evolutionary theories of aging have suggested that delaying reproductive time can prolong life span. Previous studies have identified that late fertility had positive effects on post-reproductive survival (35–40). This study indicated that ICA and LCA were significantly higher in centenarian women than in women aged 80–99 years. In late pregnancy, the mother benefits from sharing blood with the fetus, with the recovery of aging organs due to the physiological changes in late childbearing and breastfeeding.

It is generally believed that E2 and T levels are beginning to rise at puberty and reach to the top at the age of 30–40 years. As people get older, E2 and T levels continue to decline faster and faster. It is still unclear what role E2 and T play in oldest-old population. This study suggested that E2 levels had a positive association with age and longevity, and serum T levels had a negative association with age and longevity in Chinese oldest-old population. High levels of endogenous estrogen at menopause can prevent or delay the onset of aging, cardiovascular and other disease (41, 42). The net effect of late menopause is usually increased life span. Additionally, microarray deoxyribonucleic acid analyses of women aged over 45 years have shown that genes related to delayed ovarian aging were also involved in successful aging (43). Thus, late menopause generally indicates a long life span (44–46).

The current study had some limitations. Firstly, age at menopause was not taken into account in this study. It was very difficult for oldest-old individuals to recall age at menopause or obtain this information from their friends, relatives, offspring, or population survey/medical records. Secondly, this cross-sectional study had no long-term follow-up to determine deceased women. We actually do not know if women aged 80–99 years will be centenarian women in a few years.

## Conclusion

Based on the data from the CHCCS, this study demonstrated that the centenarians had crucial characteristics of less and delayed childbearing, indicating a negative relationship between female fertility and longevity, in Chinese oldest-old population. Meanwhile, serum E2 levels were positively associated and serum T levels were negatively associated with longevity. The less and late childbearing might be a significant factor of prolonged life span, and centenarian longevity and successful aging might be promoted by reducing and delaying female childbearing.

## Data Availability Statement

The raw data supporting the conclusions of this article will be made available by the authors, without undue reservation.

## Ethics Statement

The studies involving human participants were reviewed and approved by the Ethics Committee of Hainan Hospital of Chinese People’s Liberation Army General Hospital (Sanya, Hainan; Number: 301hn11201601). The patients/participants provided their written informed consent to participate in this study.

## Author Contributions

QiaoZ, SF, QianZ, JT, YZ, and YY contributed to the study design, conducted the data collection and analyses, and drafted the paper. All authors contributed to the article and approved the submitted version.

## Funding

This work was supported by grants from the National Natural Science Foundation of China (81900357, 81903392, 81941021, 81901252, and 82001476), the Military Medical Science and Technology Youth Incubation Program (20QNPY110), the National Key R&D Program of China (2018YFC2000400), the National S&T Resource Sharing service platform Project of China (YCZYPT[2018]07), the General Hospital of PLA Medical Big Data R&D Project (MBD2018030), the China Postdoctoral Science Foundation funded project (2019M650359), the National Geriatric Disease Clinical Medicine Research Center Project (NCRCG-PLAGH-2017-014), the Central Health Care Scientific Research Project (W2017BJ12), the Hainan Medical and Health Research Project (16A200057), the Sanya Medical and Health Science and Technology Innovation Project (2016YW21, 2017YW22, and 2018YW11), the Military Medicine Youth Program of Chinese PLA General Hospital (QNF19069), and the Clinical Scientific Research Supporting Fund of Chinese PLA General Hospital (2017FC-CXYY-3009). The sponsors had no role in the design, conduct, interpretation, review, approval, or control of this article.

## Conflict of Interest

The authors declare that the research was conducted in the absence of any commercial or financial relationships that could be construed as a potential conflict of interest.
